# Therapeutic impact of Mongolian Andai therapy on gut microbiota and metabolomic profiles in depression

**DOI:** 10.3389/fnins.2026.1743844

**Published:** 2026-06-03

**Authors:** Qila Sa, Miao Mei, Yuanyuan Ma, Yunfei Niu, Murilige Chao, Yagetu Hu, Yumei Qi

**Affiliations:** 1 Ethnic Medicine Innovation Center, Inner Mongolia Medical University, Hohhot, Inner Mongolia, China; 2 Five-Therapy Rehabilitation Department, Inner Mongolia International Mongolian Medicine Hospital, Hohhot, Inner Mongolia, China; 3 Mongolian Medicine Department, Affiliated Hospital of Inner Mongolia Medical University, Hohhot, Inner Mongolia, China; 4 Affective Disorder Treatment Department, Inner Mongolia Mental Health Center, Hohhot, Inner Mongolia, China; 5 Hematology Department, Inner Mongolia International Mongolian Medicine Hospital, Hohhot, Inner Mongolia, China

**Keywords:** Andai therapy, depression, gut microbiota, metabolomics, treatment response

## Abstract

**Introduction:**

Depression remains a leading global health issue, with many patients failing to respond to traditional treatments. Emerging research links depression to gut microbiota dysbiosis, suggesting a role for microbiota-based interventions. This study explores the effects of Mongolian Andai therapy on gut microbiota and metabolites in depressed patients.

**Methods:**

Twenty patients with depression underwent 8 weeks of Andai therapy. Gut microbiota was analyzed using 16S rRNA sequencing, and blood metabolites were analyzed through metabolomics. Bioinformatic analyses, including PCA and KEGG enrichment, were performed to identify treatment-related changes.

**Results:**

Andai therapy resulted in significant changes in gut microbiota, including an increase in *Proteobacteria* and a decrease in Bacteroidota in the improved patients. Metabolomic analysis revealed alterations in pyrimidine and purine metabolism, with key genera such as Escherichia-Shigella and Bacteroides linked to treatment response. Integrated analysis identified microbial-metabolite associations associated with therapeutic efficacy.

**Conclusion:**

Andai therapy modulates gut microbiota and metabolic pathways related to depression, suggesting its potential as a complementary treatment. Larger studies and multi-omics approaches are necessary to explore these mechanisms further.

## 1 Introduction

Depression is a prevalent and life-threatening psychiatric disorder characterized by persistent low mood, anhedonia, sleep and appetite disturbances, cognitive dysfunction, and in severe cases, suicidal tendencies (Moussavi et al., 2007; Simon, 2003). The World Health Organization (WHO) estimates that 5% of adults worldwide suffer from depression, affecting approximately 300 million people (Nagy et al., 2020). By 2030, depression is expected to be the leading cause of global disease burden due to premature mortality and years lived with disability (Funk, 2016). Depression is a heterogeneous disorder with incompletely understood pathophysiology. Current evidence suggests multifactorial pathological mechanisms, including dysregulation of monoamine neurotransmitters (MNTs) and their receptors, hypothalamic-pituitary-adrenal (HPA) axis dysfunction, genetic and epigenetic anomaly, chronic inflammation, impaired neurotrophic signaling, and the social psychological hypothesis (Cui et al., 2024; Malhi and Mann, 2018; Milaneschi et al., 2020; Stetler and Miller, 2011). However, none of these factors alone can fully explain the pathological basis of depression. Furthermore, despite the availability of treatment, more than half of patients do not respond to initial antidepressant therapy, and a large proportion fail to achieve remission even after multiple treatment attempts, defining treatment-resistant depression (TRD) (Howland, 2008). Therefore, a deeper understanding of the underlying mechanisms and the development of novel, effective therapeutic strategies are urgently needed to improve the clinical outcomes of depression.

Accumulating evidence has suggested the correlation between depression and microbiota-gut-brain (MGB) axis dysfunction (Liang et al., 2018; Naseribafrouei et al., 2014; Winter et al., 2018). Compared to healthy controls, major depressive disorder (MDD) patients demonstrated significant alterations in gut microbiota composition, including increased abundance of *Bacteroidetes* (Chung et al., 2019; Jiang et al., 2015; Yang et al., 2020), *Actinobacteria* (Chung et al., 2019; Jiang et al., 2015), and *Proteobacteria* (Jiang et al., 2015), alongside reduced abundance of *Lactobacillus* (Aizawa et al., 2016), *Bifidobacterium* (Aizawa et al., 2016; Zheng et al., 2020), *Firmicutes* (Jiang et al., 2015; Zheng et al., 2016), and *Lachnospiraceae* (Naseribafrouei et al., 2014). The relationship between the MGB axis and depression is further elucidated by experimental evidence that fecal microbiota transplantation from MDD patients to rodents induces depression-like behaviors (Cryan et al., 2019; Kelly et al., 2016). These findings highlight the therapeutic potential of targeting the gut microbiota in depression treatment, with emerging clinical studies supporting microbiota-based interventions. For instance, clinical preliminary data have reported the efficacy of adjunctive therapy combining *Clostridium butyricum* CBM588 with conventional antidepressants in TRD patients (Miyaoka et al., 2018). In addition, fecal 16S rRNA sequencing analysis suggested that gut microbiota restoration may contribute to the rapid and sustained antidepressant effects of the N-methyl-D-aspartate receptor (NMDAR) antagonist ketamine in TRD (Qu et al., 2017). Recent studies have suggested that AI-assisted microbiome analysis may facilitate biomarker discovery in depression (Lin et al., 2025). Strain-specific probiotic strategies have also attracted increasing attention as potential psychobiotic interventions for depressive symptoms (Rahmannia et al., 2024). In addition, fecal microbiota transplantation (FMT) has recently emerged as another microbiota-targeted approach in depression research (Zhang et al., 2025). Stress-induced gut microbiota dysbiosis in MDD triggers systemic inflammation, characterized by elevated IL-6/IFN-γ and reduced short-chain fatty acids, while compromising gut barrier integrity (Sampson et al., 2016; Skonieczna-Żydecka et al., 2018; Zhang et al., 2017). These microbiota-derived proinflammatory cytokines and neurotoxins cross the blood-brain barrier to trigger neuroinflammation and glial dysfunction (Pan et al., 2014), which subsequently activates the kynurenine pathway and tryptophan metabolism. This metabolic shift promotes the production of tryptophan catabolites, including the NMDAR agonist quinolinic acid (QUIN), which influences glutamate transmission and ultimately contributes to the pathogenesis of depressive symptom (Woodburn et al., 2021; Zhang et al., 2020).

Regarding the relationship of gut microbiota with the host metabolic phenotype, the metabolomic profiling can disclose aberrant metabolites and metabolic pathways involved in complex diseases, including depression (de Kluiver et al., 2023). Metabolomic analyses have demonstrated evidence of disrupted metabolic pathways, particularly those involved in amino acid metabolism, acetate degradation, unsaturated fatty acid metabolism, fatty acid biosynthesis, and the biosynthesis of neurotransmitter precursors (Du et al., 2021; Jiang et al., 2024; Jianguo et al., 2019). Reduced levels of metabolites such as tryptophan, its derivative serotonin, kynurenine, and related indole compounds have been linked to impaired neurotransmission and the development of depressive symptoms (Dantzer, 2016; Lukić et al., 2022; Oxenkrug, 2013). Moreover, decreased production of SCFAs, such as acetate, propionate, and butyrate, has been frequently observed and is believed to compromise gut barrier integrity and anti-inflammatory signaling, thus exacerbating neuroinflammation (Cheng et al., 2024; Xiao et al., 2022). Intriguingly, deficiencies in neuroactive substances such as vitamins (B6, B12, riboflavin, folate) have been implicated in depressive pathophysiology (Murakami et al., 2010). These findings position microbiota-modulated metabolic disruptions as one of the central mechanisms bridging gut dysbiosis to depression, offering novel targets for therapeutic intervention.

Andai therapy represents a unique integration of traditional Mongolian healing arts, combining psychosomatic regulation through rhythmic dance, therapeutic music, and structured movement therapy (Saijirahu, 2008; Sharina, 2017). Recognized for its cultural and medical significance, this practice was officially listed as a National Intangible Cultural Heritage by the Chinese State Council in 2006 (Sa et al., 2022). Although historical records documented Andai’s therapeutic applications for various conditions (Sa et al., 2022; Sharina, 2017), contemporary research has predominantly focused on literature, art, sociology, and performance studies, with limited attention to its potential as a therapeutic intervention. However, limited systematic research exists on Andai therapy’s therapeutic mechanisms and clinical applications in depression.

In this study, we employed an integrative approach combining 16S rRNA sequencing and metabolomics profiling to systematically investigate the therapeutic effects of Andai therapy on gut microbiota composition and metabolic signatures in depressed patients. By characterizing microbial and metabolic alterations pre- and post-intervention, we aim to elucidate the potential mechanisms underlying the antidepressant effects of Andai therapy and identify novel targets for depression treatment strategies. Furthermore, we seek to establish gut microbiome and metabolite biomarkers that could facilitate patient stratification, enabling the identification of individuals who may derive maximal benefit from this traditional Mongolian therapy, thereby paving the way for personalized treatment approaches in depression management.

## 2 Materials and methods

### 2.1 Study subjects

#### 2.1.1 Enrolled patients

Patients were selected from the international Mongolia Hospital of Inner Mongolia from January 2021 to June 2022. The Hamilton Depression Rating Scale–24 (HAMD–24) was used to quantitatively assess patients’ depressive symptoms. The following criteria were used to assess the severity of depression: no depression (0–7); mild depression (8–16); moderate depression (17–23); and severe depression ( ≥ 24) (Zimmerman et al., 2013). According to the predefined inclusion and exclusion criteria, we enrolled patients with depression who underwent Mongolian medical Andai therapy as study subjects. The patients received only Andai therapy intervention, which was performed by experienced Mongolian medical practitioners following standardized operating procedures, with a treatment course of 8 weeks. Clinical efficacy was evaluated using the HAMD. Responders were defined as those with a ≥ 50% reduction in HAMD scores after 8 weeks of treatment, while non-responders did not meet this criterion. Based on treatment efficacy, 20 participants were categorized into responder and non-responder groups. The study protocol was approved by the Ethics Committee of Inner Mongolia Medical University (Approval No. YKD202102127), and informed consent was obtained from all enrolled patients.

#### 2.1.2 Inclusion criteria

Patients meeting the diagnostic criteria for depression according to the “ICD-11 Clinical Descriptions and Diagnostic Guidelines for Mental and “Behavioral Disorders” (First et al., 2015); First-episode depression with HAMD-24 score ≥ 8; Age ≥ 18 years; No history of substance abuse or alcohol dependence; No severe suicidal tendencies; Able to understand and cooperate with scale assessments; Provided informed consent.

#### 2.1.3 Exclusion criteria

Patients with secondary depression caused by other etiologies (e.g., hypothyroidism, drug side effects); Age <18 or >60 years; Pregnant or breastfeeding women; Patients who had taken antidepressant medications; Patients unable to cooperate with required examinations or scale assessments.

#### 2.1.4 Withdrawal criteria

Participants who withdrew during the study; Poor compliance.

### 2.2 16 S rRNA gene sequencing

#### 2.2.1 Fecal sample collection and preservation

Fresh fecal specimens were collected from all participants at both baseline (enrollment) and post-treatment time points. To ensure nucleic acid stability, samples were immediately placed in sterile collection tubes containing DNA stabilization buffer, allowing temporary room-temperature storage prior to long-term preservation at −80°C.

#### 2.2.2 Fecal DNA extraction and PCR amplification

Total genomic DNA was extracted from fecal samples using the QIAamp PowerFecal Pro DNA Kit (Qiagen, Germany) following the manufacturer’s protocol. DNA concentration and purity were quantified using a NanoDrop 2000 spectrophotometer (Thermo Fisher Scientific, United States), while integrity was verified by 1.5% agarose gel electrophoresis. The V3-V4 hypervariable regions of the bacterial 16S rRNA gene were amplified using universal primers 343F (5′-TACGGRAGGCAGCAG-3′) and 798R (5′-AGGGTATCTAATCCT-3′), which included Illumina sequencing adapters and a sample-specific barcode on the reverse primer. The PCR products were then examined and extracted from 2% agarose gels.

#### 2.2.3 Library construction and sequencing

DNA was mechanically fragmented to an average fragment size of 350 bp using the Covaris M220 focused-ultrasonicator (Covaris LLC, Woburn, MA, United States). 16S rRNA gene sequencing libraries were prepared following the manufacturer’s protocols with the TruSeq DNA PCR-Free Sample Preparation Kit (Illumina, San Diego, CA, United States). Library quality was evaluated using a Qubit^®^ 2.0 Fluorometer (Thermo Fisher Scientific, Waltham, MA, United States) and quantitative PCR. Then, paired-end sequencing (PE 250 bp) was conducted on the NovaSeq 6000 platform (Illumina) at OE Biotech Company (Shanghai, China).

#### 2.2.4 Bioinformatic analyses

Raw sequencing reads were preprocessed using Cutadapt 4.9 to remove adapter sequences. Subsequent analysis was performed in Quantitative Insights in Microbial Ecology 2 (QIIME2) (2020.11) (Bolyen et al., 2019) using the DADA2 (Callahan et al., 2016) plugin with default parameters, which included quality trimming, denoising, read merging, and chimera removal. The output included the representative reads and an amplicon sequence variant (ASV) abundance table for taxonomic annotation. The representative reads of each ASV were selected using the QIIME2 package. Taxonomic annotation was performed using the q2-feature-classifier plugin against the SILVA database (v138)^1^ (for 16S/18S rDNA) or UNITE (for ITS),^2^ with default classification thresholds.

The final homogenized sequence data were used to evaluate microbial diversity using both alpha- and beta-diversity metrics. Alpha diversity was assessed via community richness (Chao1 and ACE indices), diversity (Shannon and Simpson indices), and phylogenetic diversity (PD_whole_tree). Beta diversity was calculated using Unweighted UniFrac distances and visualized through principal component analysis (PCA; R package factoextra v1.0.7). To examine community-environment relationships, redundancy analysis (RDA; R package vegan v2.6-8) was applied. Group differences in alpha-diversity indices were tested using Student’s *t*-tests and illustrated with boxplots. Taxonomic composition was summarized by displaying the top 10 abundant families and genera (“Others” for remaining taxa) and plotted as stacked bar plots. Differential taxa across groups were identified via LEfSe (R package microeco v1.11.0; LDA score > 2.0 (Segata et al., 2011)), and KEGG pathway abundance variations were analyzed using *t*-tests (*p* < 0.05 was considered statistically significant).

#### 2.2.5 Public dataset collection and external validation

To validate the reproducibility of the microbiota features identified in our cohort, a publicly available 16S rRNA sequencing dataset (PRJNA591924) was downloaded from the NCBI Sequence Read Archive. This dataset was used as an independent external cohort for validation. Genus-level relative abundance data were extracted, and selected genera of interest were compared between the major depressive disorder and healthy control groups using the same analytical framework applied to our cohort.

### 2.3 Metabolomics analysis

#### 2.3.1 Sample preparation

Fasting blood samples were collected in EDTA anticoagulant tubes at baseline and post-treatment intervals, followed by immediate plasma separation and storage at −80°C until subsequent analysis. For LC-MS-based metabolomic analysis, thawed samples were mixed with L-2-chlorophenylalanine (0.06 mg/mL in methanol) an internal standard and extracted with ice-cold methanol/acetonitrile (2:1, v/v) using ultrasonication in an ice-water bath. Following protein precipitation at −20 °C for 30 min and centrifugation (13,000 × g, 4°C, 10 min), the supernatant was concentrated by freeze concentration centrifugal dryer. The residue was reconstituted in methanol/water (1:4, v/v), vortexed, and subjected to secondary extraction by ultrasonication on ice, followed by incubation at −20°C for 2 h. After final centrifugation (13,000 × g, 4°C, 10 min), the supernatant was filtered through 0.22 μm membranes into LC vials and stored at −80°C until analysis. Quality control samples were prepared by pooling equal aliquots from all experimental samples.

#### 2.3.2 LC-MS/MS analysis

Metabolomic analysis was conducted by Shanghai Luming Biological Technology Co., Ltd (Shanghai, China) using a high-resolution LC-MS platform consisting of an ACQUITY UPLC I-Class Plus system (Waters Corporation, Milford, United States) interfaced with a Q-Exactive hybrid quadrupole-Orbitrap mass spectrometer (Thermo Fisher Scientific, Waltham, MA, United States). The system was operated in dual-polarity mode with a heated electrospray ionization (H-ESI) source to ensure comprehensive metabolite coverage (H-ESI positive and negative ion modes). For chromatographic separation, an ACQUITY UPLC HSS T3 column (1.8 μm, 2.1 × 100 mm) was employed under optimized conditions to achieve high-resolution separation of complex metabolic components.

#### 2.3.3 Data processing and bioinformatics analysis

Raw LC-MS data were processed using Progenesis QI V2.3 (Nonlinear, Dynamics, Newcastle, United Kingdom) for baseline correction, peak picking (5 ppm precursor mass tolerance), retention time alignment, and normalization. Metabolite identification was performed by matching accurate mass (10 ppm product ion tolerance) and MS/MS fragmentation patterns against Human Metabolome Database (HMDB),^3^ LipidMaps (v2.3),^4^ METLIN,^5^ and in-house databases. Data quality control included: (a) removal of peaks with missing values (intensity = 0) in over 50% of samples across groups, (b) imputation of zeros with half-minimum values, and (c) exclusion of compounds with identification scores < 36/60. Positive and negative mode datasets were merged into a unified matrix for subsequent statistical analysis (Xu et al., 2024).

The merged data matrix was analyzed in R using PCA to assess sample distribution and process stability. Metabolites were ranked by variable importance in projection (VIP) scores from OPLS-DA, with those exhibiting VIP > 1.0 and *p* < 0.05 (two-tailed Student’s *t*-test) considered statistically significant (Shi et al., 2025). Differential metabolites between groups were identified using Student’s *t*-test, with selection criteria of fold change (FC) ≥ 1.2 and *p* < 0.05. Pathway enrichment analysis of differential metabolites was performed using MetaboAnalyst 6.0, with KEGG metabolic pathways as the primary reference database. Weighted Gene Co-expression Network Analysis (WGCNA) was performed using the R package “WGCNA” (v 1.73) to construct metabolite co-expression networks and identify treatment-associated modules through soft-thresholding power selection based on scale-free topology, dynamic tree cutting for module detection, module-trait relationship analysis, and preservation statistics for network robustness evaluation.

### 2.4 Integrative analysis of microbiome-metabolome interactions

To elucidate potential mechanistic links between gut microbiota composition and host metabolic profiles, we conducted Spearman’s rank correlation analysis comparing genus-level microbial abundance with serum metabolite concentrations. The results were visualized as a clustered heatmap where color gradients represent correlation coefficients (*r*-values) and hierarchical clustering reveals co-varying microbiome-metabolite modules. This integrated approach identified statistically significant (FDR-adjusted *p* < 0.05) microbe-metabolite associations that may reflect functional interactions within the gut ecosystem.

### 2.5 Machine learning analysis of 16S species and metabolome data

The Random Forest (RF) algorithm was used to construct binary classification models for screening potential biomarkers associated with therapeutic efficacy, with 16S species abundance and metabolome abundance as predictive variables and post-treatment efficacy groups (improve/not_improve) as target variables. RF, as an ensemble learning method based on multiple decision trees, can evaluate the contribution of each species or metabolite to the classification task. The model parameters were set as follows: the number of trees (ntree) was 500, the number of variables randomly sampled at each split (mtry) was 15, and samples with missing values were excluded. Feature importance was assessed using MeanDecreaseAccuracy (MDA) and MeanDecreaseGini (MDG).

To evaluate the stability of the top-ranked features, leave-one-out cross-validation (LOOCV) was additionally performed. In each iteration, one sample was excluded and the remaining samples were used to train the RF model and rank feature importance. The stability of the selected top features was assessed according to their consistency across LOOCV iterations.

All analyses were performed in R (version 4.3.0), using the randomForest package for model construction and feature importance estimation.

In addition, an exploratory deep learning analysis was performed using TRPCA (Myers et al., 2025), a transformer-based framework proposed for microbiome modeling. The model integrates normalized transformer layers with robust principal component analysis-oriented feature learning and supports single-task and multi-task learning. In this study, transposed genus-level abundance profiles were used as input features, and treatment-response labels (improve/not_improve) were used for supervised classification.

## 3 Results

### 3.1 Impact of Andai therapy on gut microbiota in depression patients

**FIGURE 1:**
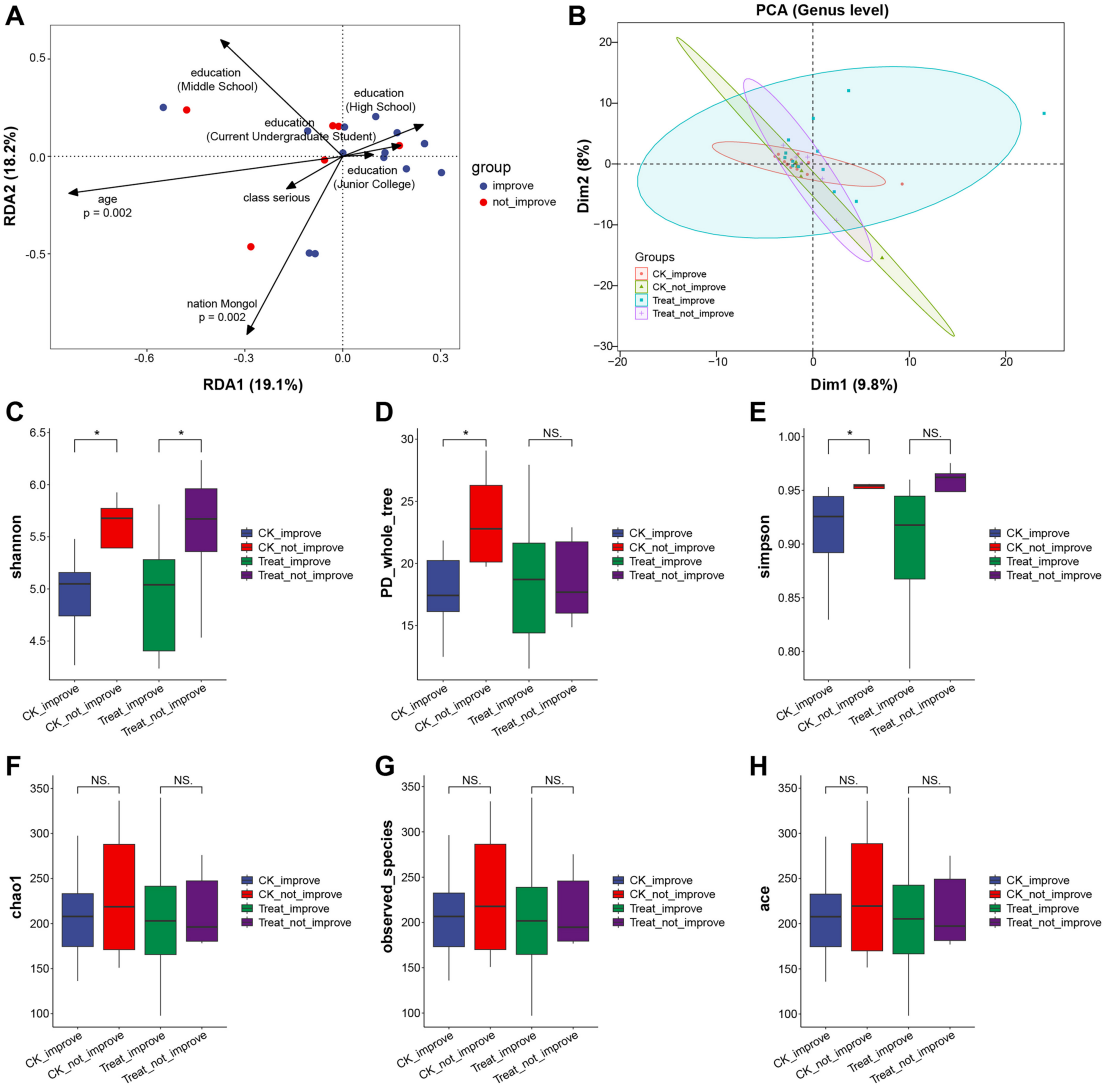
Results of 16S rRNA gene sequencing analysis. **(A)** Redundancy analysis (RDA) of 16S rRNA sequencing data in improved (red) a not-improved (blue) samples, constrained by significant environmental variables. RDA axes 1 and 2 collectively explained 37.3% of the total variation. Environmental variables were indicated by black arrows, with their direction and length reflecting their influence across improvement statuses. Each dot represents a single sample. **(B)** Principal component analysis (PCA) of gut microbiota composition based on 16S rRNA-derived ASV relative abundances. Blue squares and purple plus signs represented patients who showed improvement or no improvement, respectively, after Andai therapy treatment. Red circles and green triangles indicated the corresponding patients before Andai therapy treatment, classified by their subsequent improvement status. Alpha diversity analysis of microbial communities across six indices **(C–H)**: **(C)** Shannon; **(D)** PD whole-tree; **(E)** Chao1; **(F)** Simpson; **(G)** observed species; **(H)** ACE. Data represented the mean ± SD, mean values with different letters over the bars were significantly different and the symbol * indicates a statistically significant difference between groups (Student’s *t*-test, *p* < 0.05).

We collected data from 10 male and 10 female patients with depression (Supplementary Table 1), administering “Andai Therapy” to them. Prior to and following the treatment, we conducted 16S rRNA gene sequencing of their feces and blood metabolomics sequencing. Among these 20 individuals, 14 exhibited significant improvement in their condition after treatment, while six individuals showed no improvement post-treatment. Within this cohort, there was one case under 20 years old, while 12 cases were aged between 20 and 40 years, collectively accounting for 60% of the total participants. Participants over 40 years old constituted 35% of the total. Regarding educational background, 50% of the individuals had education beyond high school, 35% had junior high school education or lower, and 15% had completed high school. In terms of ethnicity, 85% of the participants identified as Han, while the remaining 15% identified as Mongolian. Before and after the treatment, we conducted 16S sequencing on their feces and blood metabolomics sequencing on their blood samples. The educational backgrounds and ages of the participants exhibited considerable variability. Initially, we conducted redundancy analysis (RDA) on the 16S rRNA gene sequencing results from 20 pre-treatment cases (CK group). RDA was employed to investigate the relationship between environmental factors and community structure. The results demonstrated that RDA1 and RDA2 collectively accounted for 37.3% of the community variation. Age was negatively correlated with RDA1 and exerted a significant impact, while educational level was negatively correlated with RDA1 and positively correlated with RDA2 (Figure 1A). Ethnicity was negatively correlated with RDA2 and also had a significant impact. In conclusion, although educational level and age may influence gut microbiota, RDA analysis results indicated that these factors were not statistically significant. Thus, their impact on gut microbiota may not be substantial (Figure 1A). Subsequently, we performed principal component analysis (PCA), which revealed that the 20 samples could be categorized into two groups based on whether there was a significant improvement in condition following treatment (Figure 1B). Therefore, we hypothesize that there may be a correlation between the composition of gut microbiota and the presence or absence of improvement post-treatment in the 16S analysis.

### 3.2 Impact of treatment efficacy on gut microbiota diversity and abundance

**FIGURE 2:**
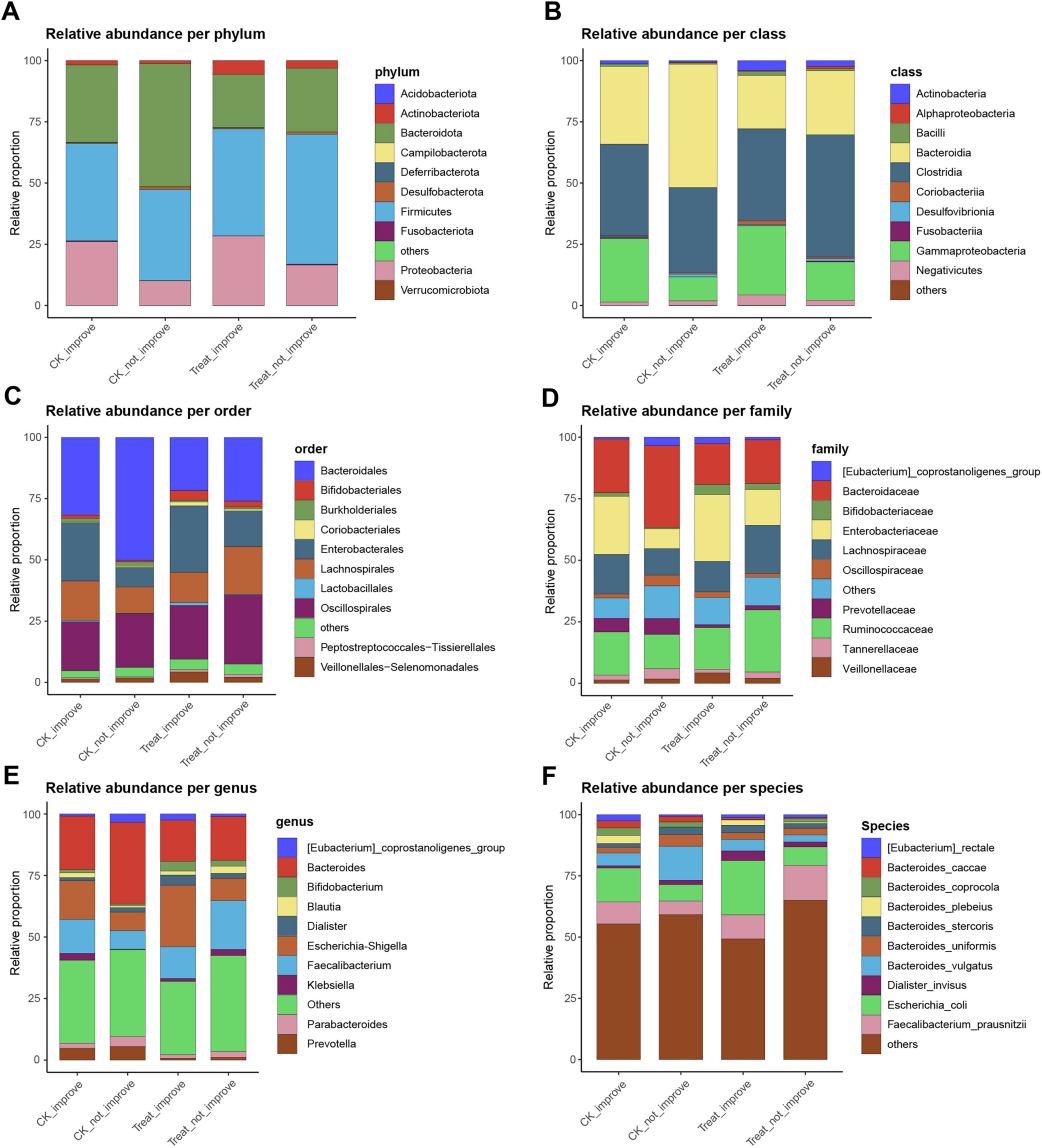
Taxonomic composition of microbial communities revealed by 16S rRNA sequencing. Stacked bars displayed the relative abundances of the top 20 most abundant taxa at six taxonomic levels: **(A-F)**: **(A)** phylum; **(B)** class; **(C)** order; **(D)** family; **(E)** genus; **(F)** species.

**FIGURE 3:**
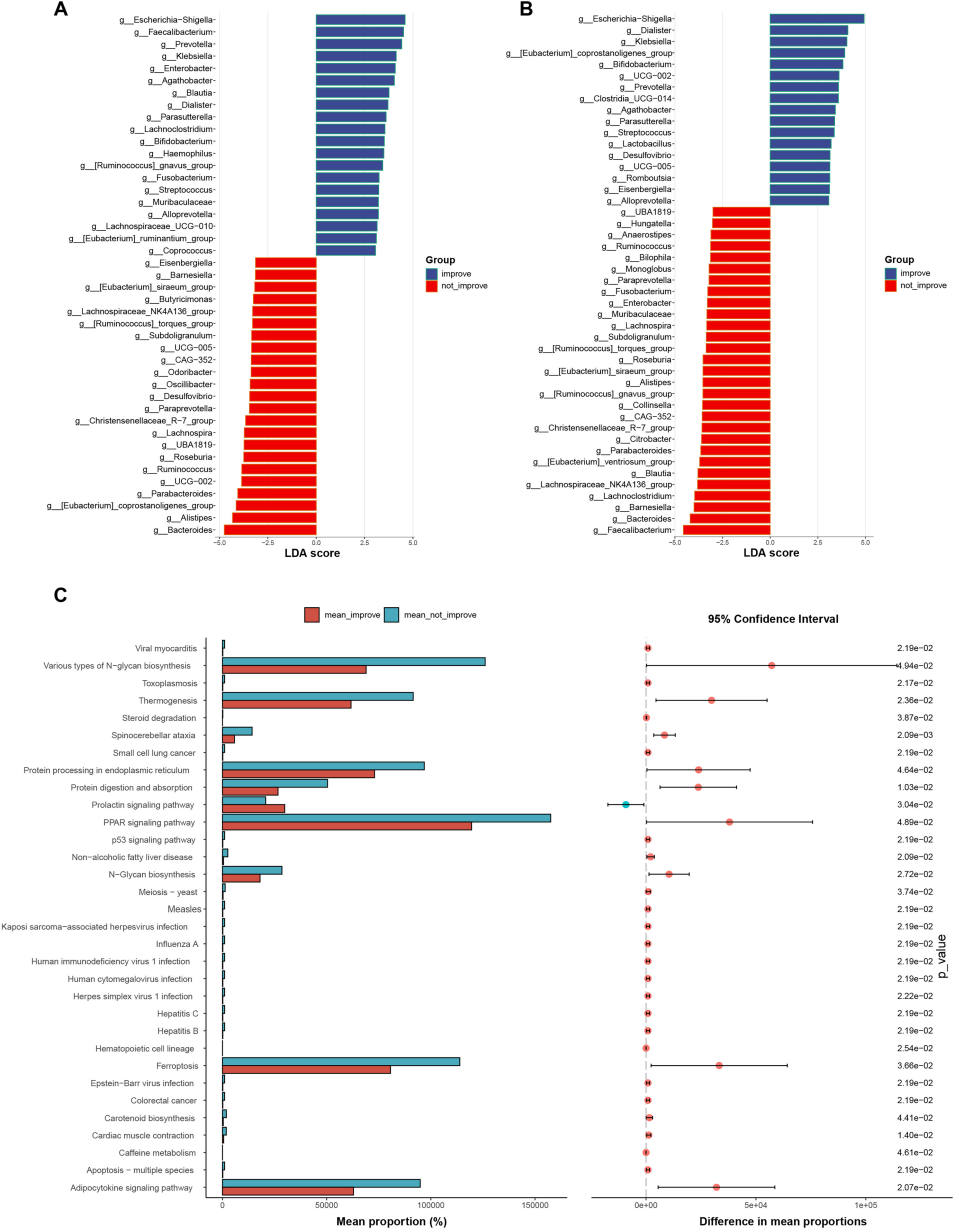
LEfSe analysis of gut microbiota biomarkers between improved and non-improved groups. **(A,B)** Genus-level differential taxa after therapy. Green bars indicated taxa enriched in the improved group (LDA score > 2), while red bars represented taxa enriched in the non-improved group (LDA score <−2). Only statistically significant biomarkers (LDA threshold |2|) were displayed. **(A)** CK group; **(B)** treat group. **(C)** KEGG enrichment analysis of differentially expressed genes between valid and invalid samples at the 16S level.

To further compare the differences in species composition between the effective and ineffective groups post-treatment, we categorized 40 16S rRNA and matched blood metabolomics samples into four groups based on treatment efficacy and before-and-after treatment: the effective group before treatment (CK-improve), the ineffective group before treatment (CK-unimprove), the effective group after treatment (Treat-improve), and the ineffective group after treatment (Treat-unimprove). First, we analyzed the differences in alpha diversity indices among these four groups based on the sequencing results of 16S (Figures 1C–H). We found an interesting phenomenon: in the CK-improve and Treat-improve groups, the Shannon and PD whole tree indices, as well as the Chao1 and Simpson indices, were all lower compared to the CK-unimprove and Treat-unimprove groups. Subsequently, we compared the differences in species abundance at different taxonomic levels among the four groups. At the phylum level, we observed that both the CK-improve and Treat-improve groups upregulated the abundance of Proteobacteria while downregulating the abundance of *Bacteroidota*. At the class level, we similarly found this consistency, with a downregulation of *Bacteroidia* abundance and an upregulation of *Negativicutes* (Figures 2A–D). This continuity persisted at both the genus and species levels (Figures 2E,F). At the genus level, this consistency can still be clearly identified in Bacteroides, Escherichia-Shigella, and *Faecalibacterium*. Although the reliability of 16S results may decrease at the species level, the findings still indicated an increase and decrease in the consistency of Bacteroides and Escherichia before and after effective treatment. Subsequently, we performed a differential analysis using LEfSe, selecting significant differences in families and genera with an LDA greater than 1. The results showed a significant difference in Escherichia in the post-treatment comparison (Figures 3A,B). We also conducted KEGG enrichment analysis at the genus level of 16S, and the results showed significant differences in pathways such as steroid metabolism, apoptosis, and ferroptosis between the improved and non-improved groups (Figure 3C). These results suggested that the effectiveness of treatment may be related to the composition of the patient’s gut microbiota, and the differences in consistency before and after treatment may indicate that the genera Escherichia and *Bacteroidia* play important roles in this process.

To assess the generalizability of the microbiota patterns identified in our cohort, we reanalyzed the public 16S rRNA dataset PRJNA591924, which includes 43 patients with major depressive disorder (MDD) and 47 healthy controls (HC). Consistent with the trends observed between the effective and ineffective groups in our Andai Therapy cohort, the external dataset showed higher relative abundances of *Bacteroides*, *Parabacteroides*, and *Prevotella* and lower relative abundances of *Faecalibacterium*, *Bifidobacterium*, and *Dialister* in the MDD group compared with HC; among these taxa, *Faecalibacterium* showed a significant decrease (Supplementary Figure 1). These concordant trends indicate that the direction of microbial alterations observed in our cohort is broadly consistent with an independent public MDD dataset, partially supporting the generalizability of our findings despite the relatively small sample size.

### 3.3 Differential metabolomic analysis of blood samples in effective vs. ineffective treatment groups

**FIGURE 4:**
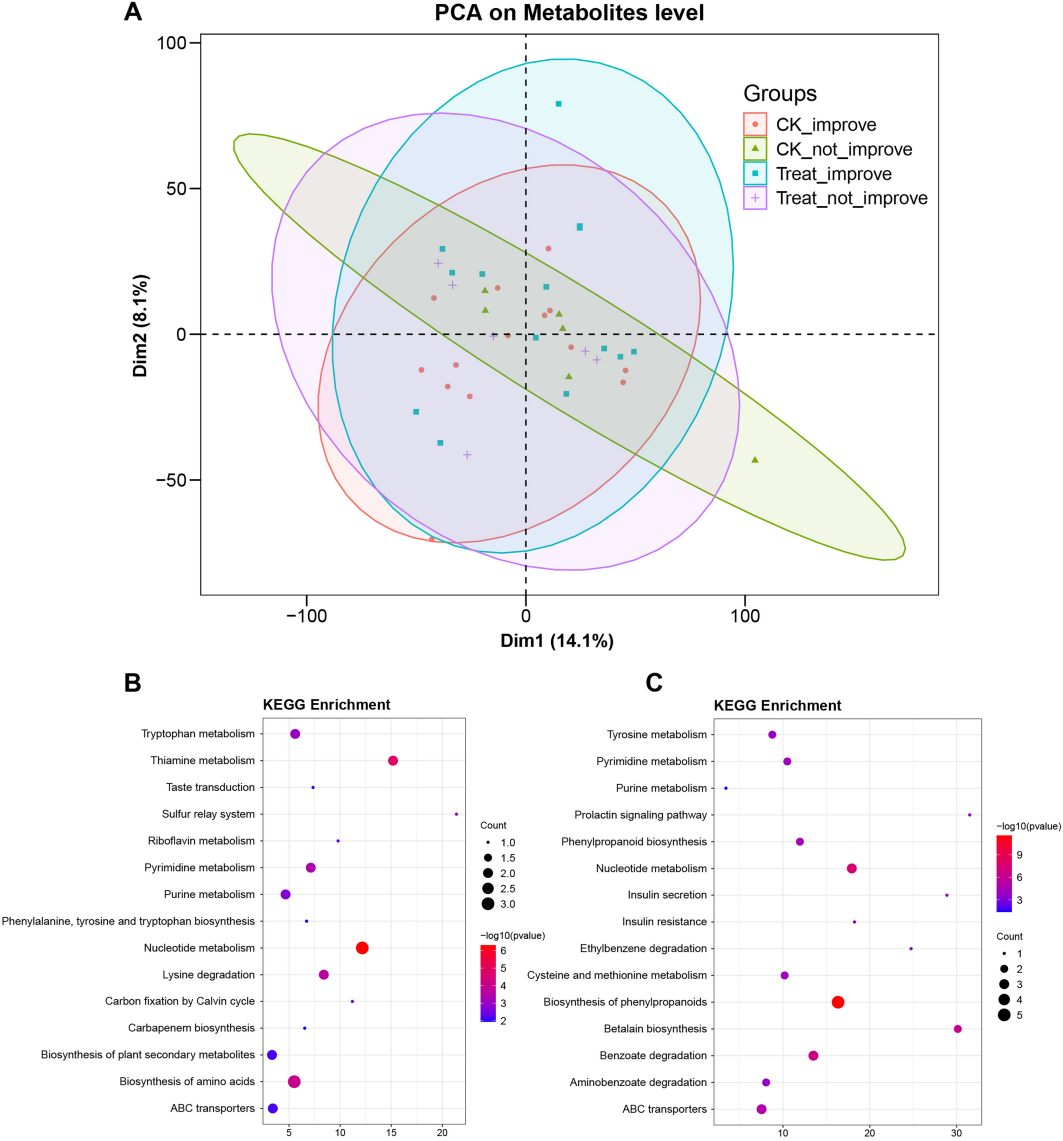
PCA and KEGG pathway enrichment analysis of metabolome. **(A)** PCA of metabolomic profiles between improved and non-improved groups before and after Andai therapy. KEGG pathway enrichment analysis of differential metabolites. **(B)** Enriched metabolic pathways before Andai therapy. **(C)** Enriched metabolic pathways after Andai therapy. Pathways were considered significantly enriched (*p* < 0.05, *t*-tests) with false discovery rate correction.

**FIGURE 5:**
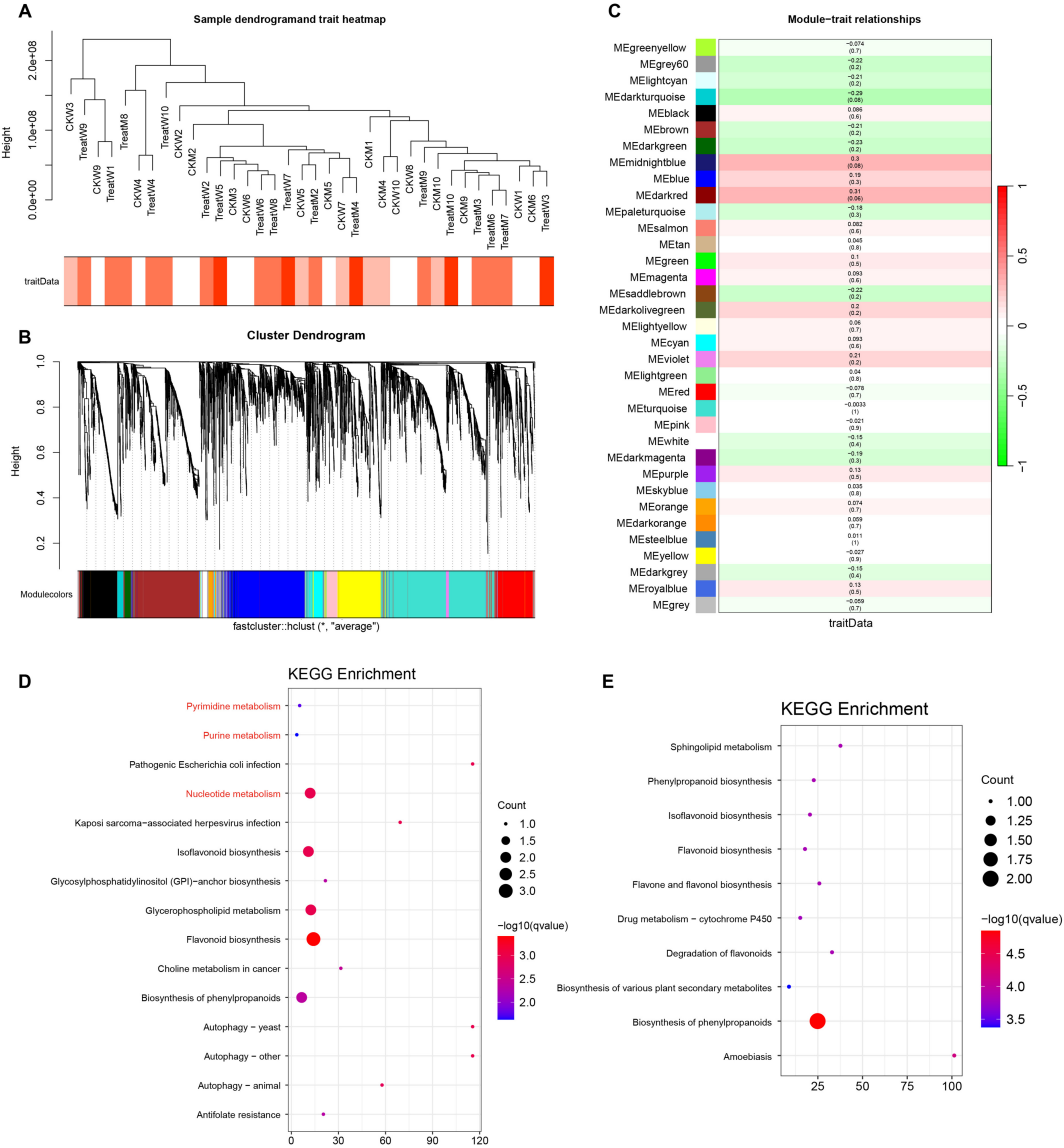
WGCNA analysis of metabolome data. **(A)** Sample clustering plot. **(B)** Hierarchical clustering dendrogram of metabolites with dynamic tree-cut module assignment (color-coded). **(C)** The correlation between clinical traits and module eigengenes. Module-trait correlations were represented by color intensity (red: positive; green: negative), with corresponding coefficients shown numerically in each cell. Corresponding p-values are shown in parentheses. **(D,E)** The KEGG enrichment analysis results corresponding to the metabolites contained in the MEmidnightblue (left) and MEdark modules (right).

To further investigate the differences between the effective and ineffective groups, we analyzed the metabolomic sequencing data of the blood. First, we performed PCA dimensionality reduction analysis on the four groups of samples (Figure 4A). The results indicated that the four groups could not be well clustered according to their classifications, but they could be loosely grouped into four major groups. Subsequently, we conducted differential analysis between the effective and ineffective groups before treatment and performed KEGG enrichment analysis on the differential metabolites. The results showed that Riboflavin metabolism and Pyrimidine metabolism were significantly enriched in the pre-treatment analysis. In contrast, Pyrimidine metabolism and Purine metabolism were significantly enriched in the comparison between the effective and ineffective groups after treatment (Figures 4B,C). To further distinguish the association between metabolites and diseases, we conducted WGCNA analysis based on the disease grading (Figures 5A–C). A total of 35 different modules were identified in the WGCNA analysis, with ME midnightblue and ME darkred had the highest scores and showed the greatest significance (0.06). Therefore, we conducted enrichment analysis for the metabolites in these two modules separately. The results indicated that ME midnightblue was also enriched in pathways related to Pyrimidine metabolism and Purine metabolism (Figures 5D,E). Taken together, these results suggested that metabolic pathways such as Pyrimidine metabolism and Purine metabolism may be related to the occurrence and treatment of diseases.

### 3.4 Association between metabolomics and 16S in treatment response: joint analysis of differential species and metabolites

**FIGURE 6:**
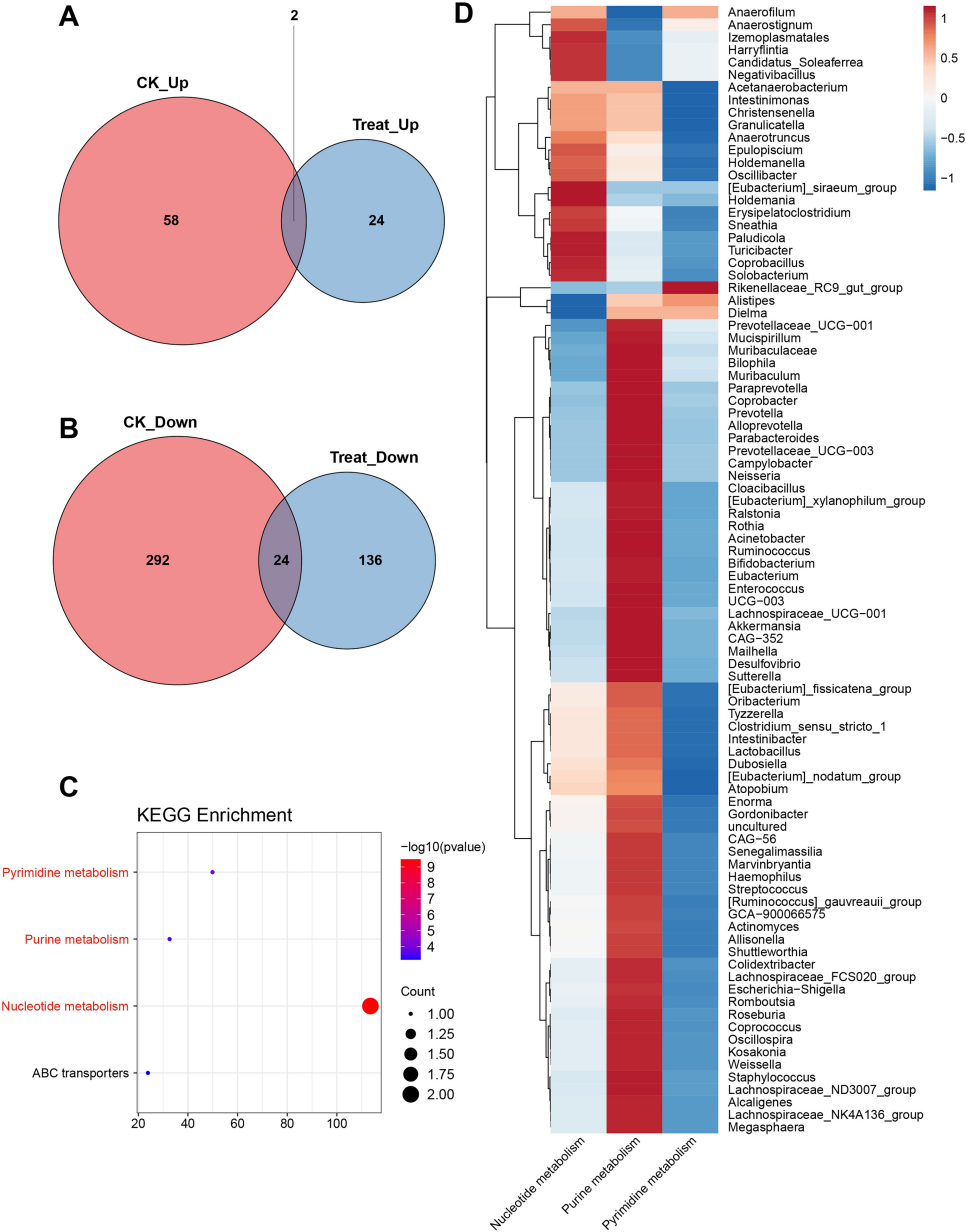
Integrated 16S rRNA sequencing of Andai therapy between improved and non-improved groups. **(A)** Venn plots of the species up-regulated in the CK group and the treat group; **(C)** Venn plots of the down-regulated species in the CK group and the treat group. **(B)** The KEGG enrichment analysis results corresponding to the 53 species with intersections in the up-regulated venn plot. **(D)** KEGG enrichment analysis results of the 38 down-regulated species corresponding to the intersection in the Venn map.

**FIGURE 7:**
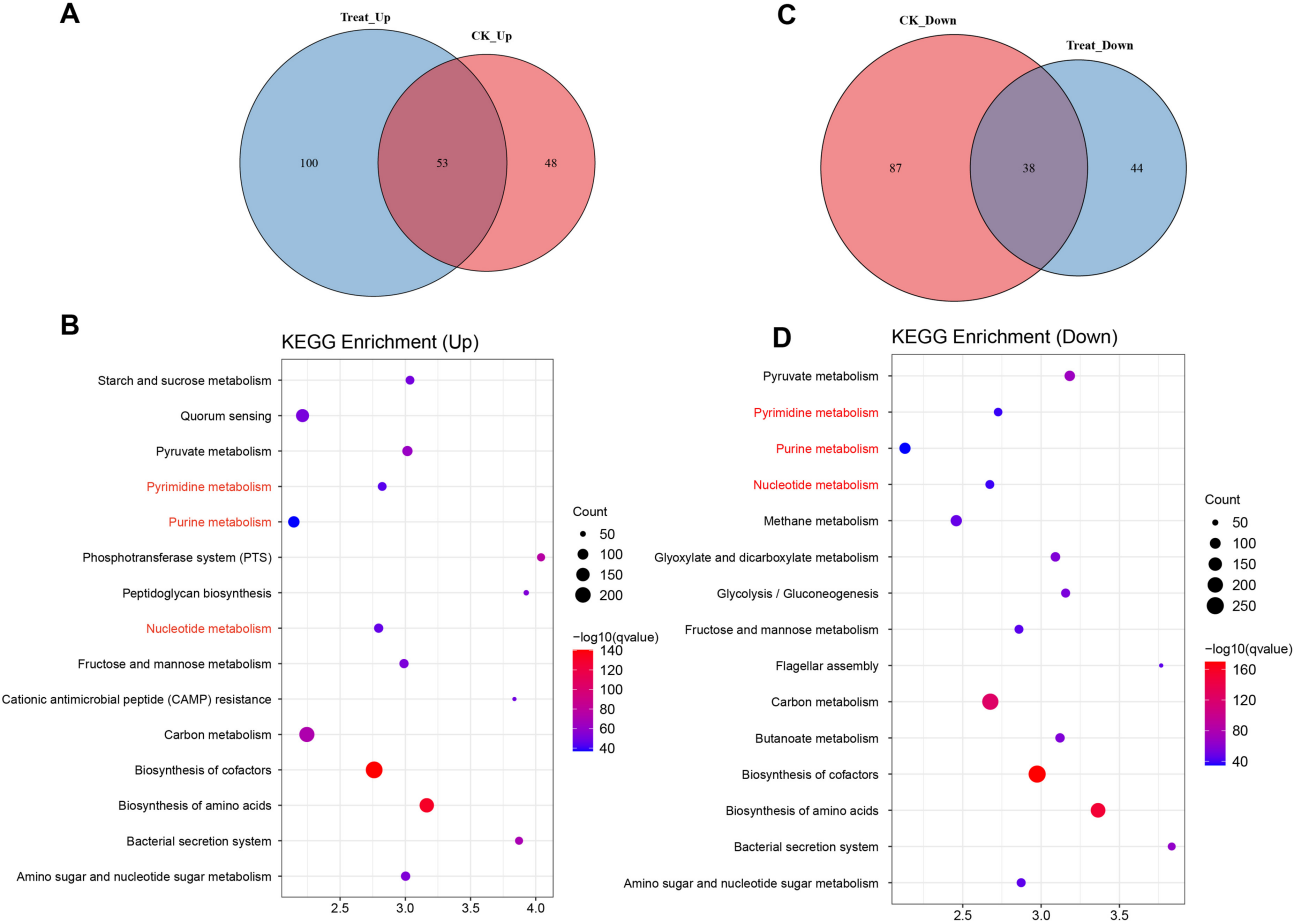
Integrated metabolomics analysis of Andai therapy between improved and non-improved groups. **(A)** Venn plots of the species upregulated in the CK group and the teat group. **(B)** Venn plots of the down-regulated species in the CK group and the treat group. **(C)** Down-regulate the KEGG enrichment analysis results of the 24 species corresponding to the intersection in the venn map. **(D)** Heatmap of correlation analysis between overlapping metabolites and bacterial genera. Rows represented genera from Venn intersections, columns showed metabolites from Venn intersections. Color scale indicated correlation strength (red: positive; blue: negative).

**FIGURE 8:**
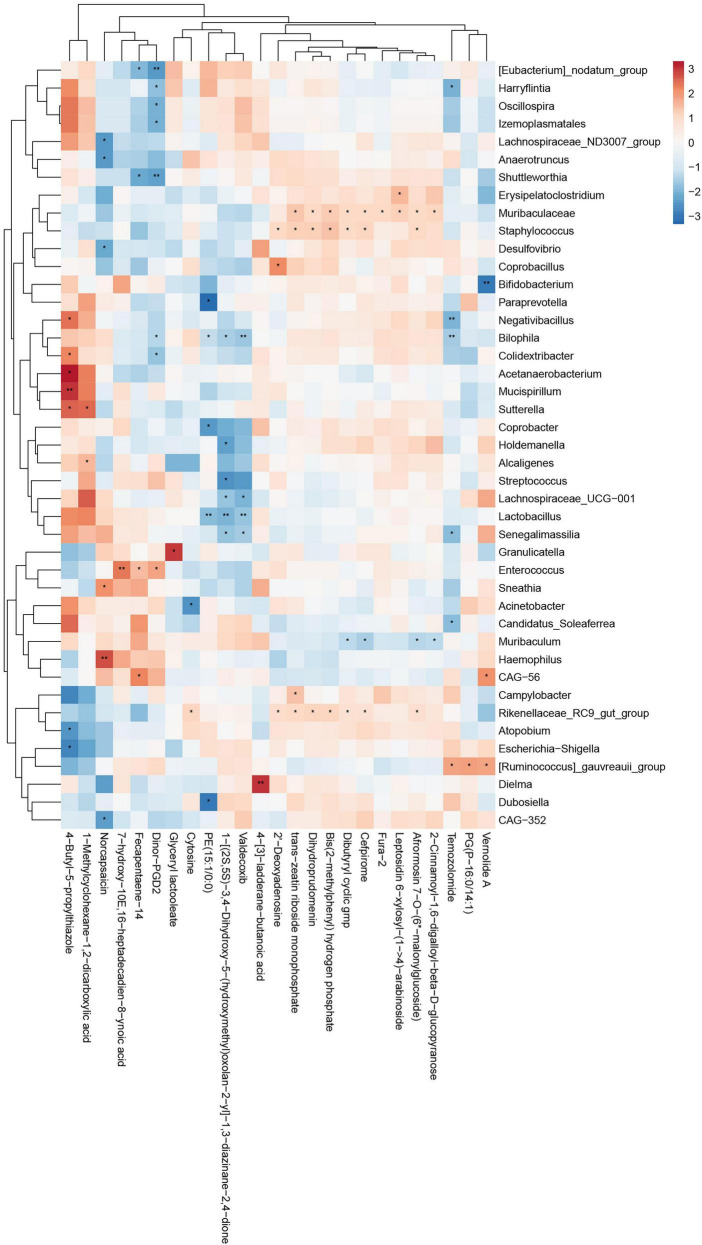
Correlation heatmap of identified microbial genera and differential metabolites. The heatmap shows Spearman correlation coefficients between the identified genera and differential metabolites. Red and blue indicate positive and negative correlations, respectively, and asterisks denote statistically significant associations. *Indicates *p* < 0.05 (statistically significant); **Indicates *p* < 0.01 (highly statistically significant).

To investigate the potential association between the metabolomics and 16S, we conducted a joint analysis of the metabolomics and 16S. Firstly, we performed statistical analysis of the differences in 16S at the genus level and the metabolomics based on two comparisons: pre-treatment and post-treatment. In the 16S results, a total of 53 genera showed consistent upregulation between pre- and post-treatment in both responder and non-responder groups (Figure 6A), while a total of 38 genera were downregulated in the intersection (Figure 6C). For the metabolomics, there were only two consistently upregulated differential metabolites and 24 consistently downregulated metabolites (Figures 7A,B). Subsequently, we performed KEGG enrichment analysis on these differential metabolites and species. By identifying the intersection of the obtained enrichment analysis results, we found that nucleotide metabolism, pyrimidine metabolism, and purine metabolism were the commonly significant differential pathways (Figures 6B,D, 7C). We then statistically analyzed the metabolites and species involved in these three pathways and found significant differences between the effective and ineffective groups. As shown in Figure 7D, the identified species exhibited distinct distribution patterns in nucleotide metabolism, purine metabolism, and pyrimidine metabolism, with several taxa showing opposite enrichment trends between purine metabolism and the other two pathways. In addition, correlation analysis between the identified species and differential metabolites revealed multiple significant positive and negative associations, and hierarchical clustering demonstrated distinct species–metabolite interaction patterns (Figure 8).

### 3.5 Identification of key pathways and microbial species in depression treatment outcomes

**FIGURE 9:**
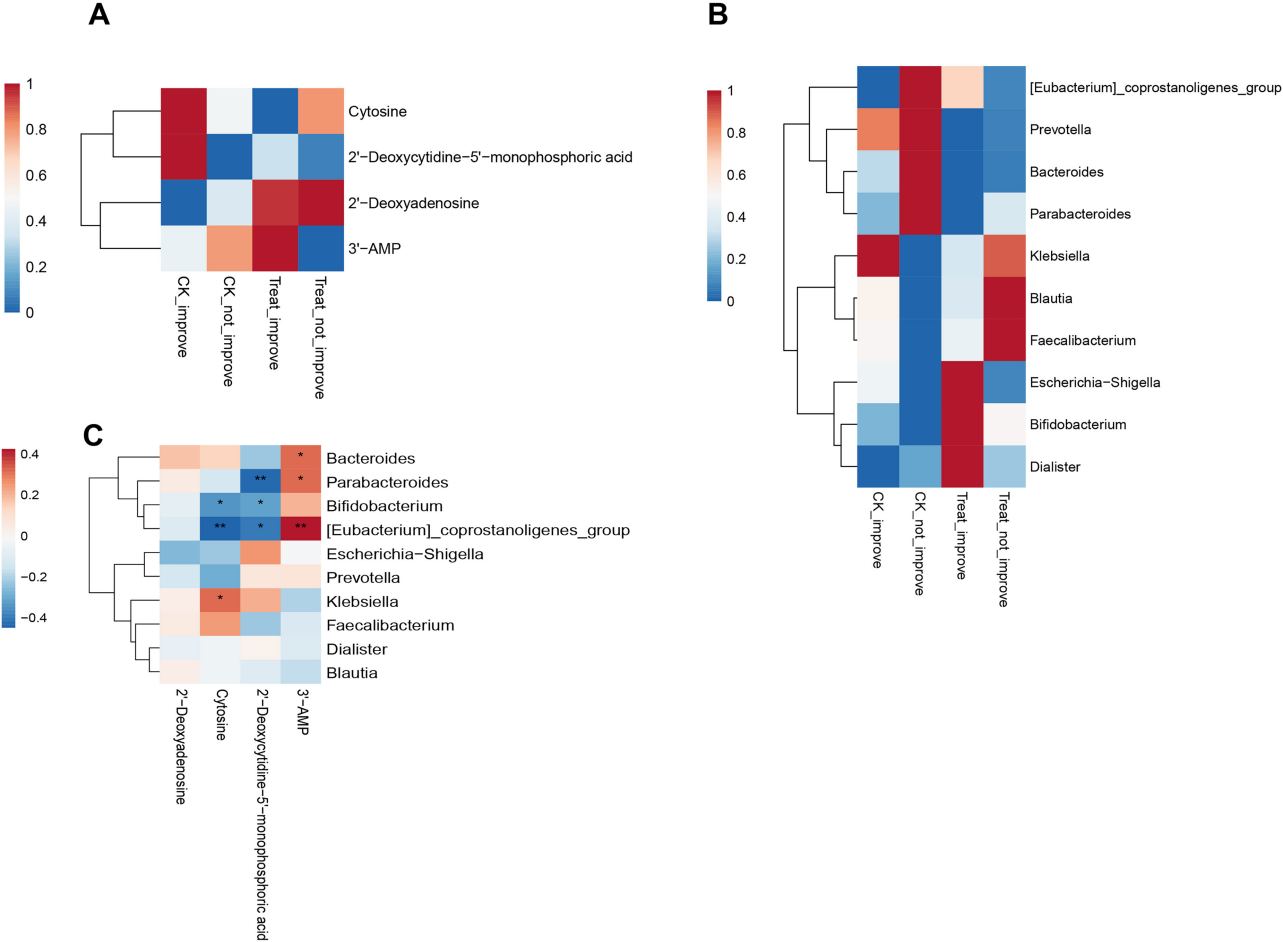
Integrated analysis of metabolites and microbial genera across four groups. **(A)** Heatmap of the intensities of metabolites involved in the intersecting pathways across the four groups. **(B)** Heatmap of the abundances of species at the 16S genus level participating in the intersecting pathways across the four groups. **(C)** Heatmap of correlation analysis between overlapping metabolites and bacterial genera. Rows represented genera from Venn intersections, columns showed metabolites from Venn intersections. Color scale indicated correlation strength (red: positive; blue: negative). *Indicates *p* < 0.05 (statistically significant); **Indicates *p* < 0.01 (highly statistically significant).

We conducted an enrichment analysis on the species and metabolites present in the intersection, subsequently selecting the common pathways derived from the results. Four pathways were identified: Pyrimidine metabolism, Purine metabolism, Nucleotide metabolism, and ABC transporters. Following this, we examined the heatmap of species involved in the enrichment of these four pathways and discovered a significant association of numerous species with Purine metabolism (Figure 9B). We also selected the differential metabolites related to the four pathways, which primarily included Cytosine, 2-Deoxycytidine-5’-monophosphoric acid, 2’-Deoxyadenosine, and 3’-AMP (Figure 9A). Furthermore, we analyzed the microorganisms significantly associated with these core metabolites. The results indicated significant correlations between Bacteroides and Parabacteroides, as well as between the Eubacterium_coprostanoligenes_group and Escherichia-Shigella (Figure 9C). Notably, these species met the criteria for the effective group, with their post-treatment abundance significantly exceeding that of pre-treatment, suggesting these microbial species and their associated metabolites contribute to depression treatements outcomes.

Taken together, these statistically significant microbial and metabolite changes were closely associated with treatment efficacy, as they converged on nucleotide-, purine-, and pyrimidine-related pathways and consistently distinguished the effective and ineffective groups. The coordinated patterns observed for key genera and metabolites suggest that these microbiota–metabolite signatures may represent biologically meaningful response-associated features of Andai therapy rather than nonspecific background variation.

### 3.6 Machine learning analysis of 16S species and metabolome data using random forest

**FIGURE 10:**
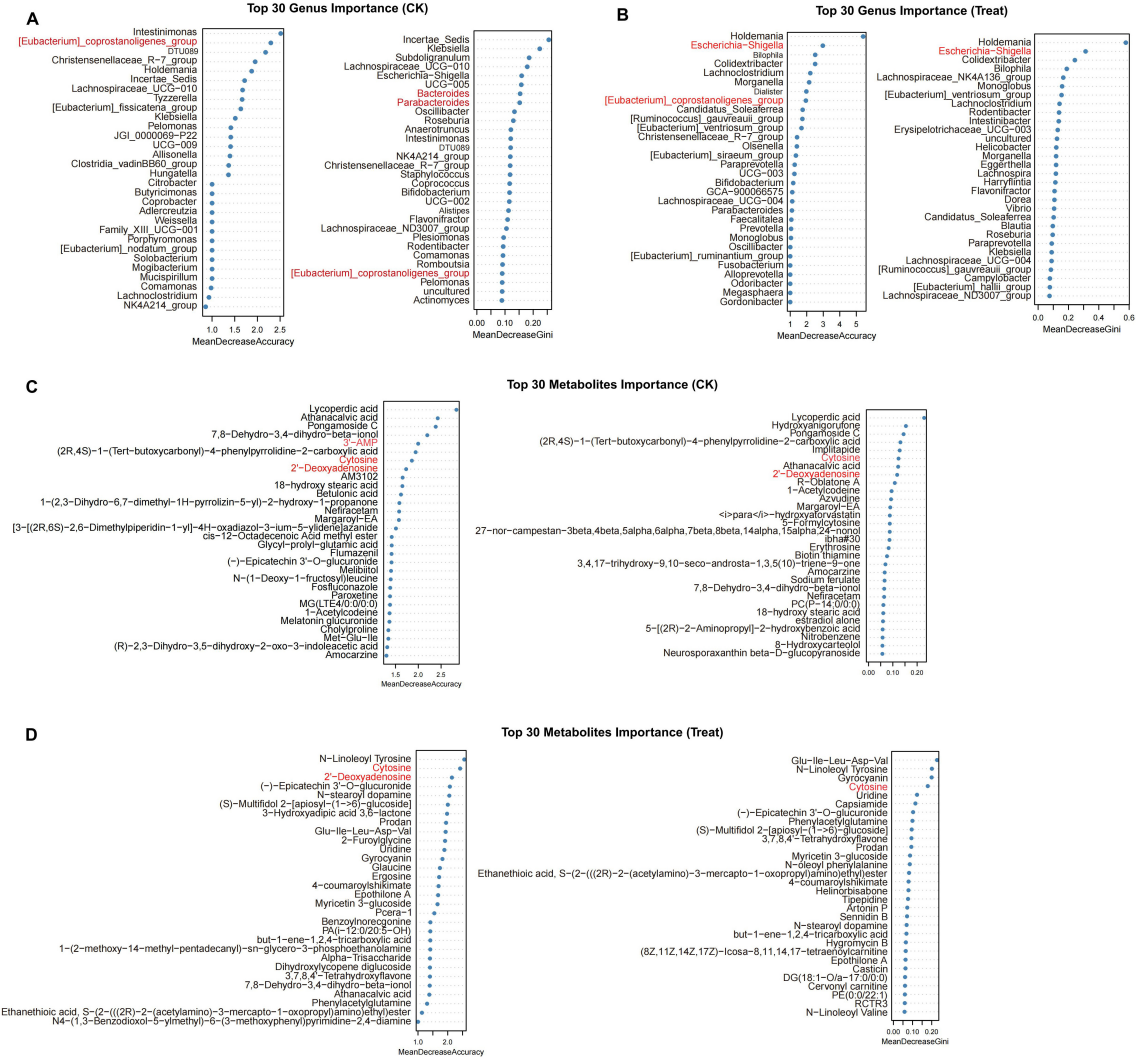
Random Forest importance analysis of 16S species and metabolome biomarkers associated with Andai therapy efficacy. **(A,B)** Top 30 genus importance visualization of gut microbiota: **(A)** CK group (before treatment). **(B)** treat group (after treatment). **(C,D)** Top 30 metabolite importance visualization: **(C)** CK group (before treatment); **(D)** treat group (after treatment).

To further validate the reliability of the results, Random Forest (RF) models were constructed based on 16S species abundance and metabolome abundance data to identify core biomarkers associated with the antidepressant efficacy of Andai Therapy, and feature importance was evaluated using MDA and MDG. In the microbial RF analysis before treatment (CK group, Figure 10A), [Eubacterium]_coprostanoligenes_group remained highly ranked, while Bacteroides and Parabacteroides also showed relatively high MDG scores, suggesting their potential relevance to efficacy differentiation. After treatment (Treat group, Figure 10B), Escherichia-Shigella ranked among the top features in both MDA and MDG, indicating a close association with post-treatment efficacy stratification, whereas [Eubacterium]_coprostanoligenes_group also maintained a relatively high importance ranking. Leave-one-out cross-validation (LOOCV) further showed that the top-ranked microbial features were more stable in the post-treatment model than in the pre-treatment model (Supplementary Table 2). In particular, Holdemania and Escherichia-Shigella were consistently retained among the top features across LOOCV iterations, while Bilophila and Colidextribacter also exhibited relatively stable rankings; in contrast, the pre-treatment model showed greater variability, although Parabacteroides remained relatively stable, especially in the MDG-based ranking. For the metabolome data, RF analyses before and after treatment are shown in Figures 10C,D, respectively. Before treatment, Cytosine and 2’-Deoxyadenosine showed relatively high MDA and MDG scores, suggesting their potential ability to distinguish subsequent therapeutic efficacy at baseline, whereas after treatment, Cytosine remained highly ranked, further supporting its close association with the efficacy of Andai Therapy. The repeated identification of taxa such as *[Eubacterium]_coprostanoligenes_group*, *Escherichia-Shigella*, *Bacteroides*, and *Parabacteroides*, together with metabolites such as cytosine and 2’-deoxyadenosine, across differential, correlation, and RF-based analyses further supports their potential clinical relevance as candidate biomarkers for treatment-response stratification.

We also performed an exploratory transformer-based analysis as a preliminary assessment of advanced deep learning approaches. Although the model achieved an apparent validation accuracy of 0.75, the training and validation curves suggested rapid convergence and unstable validation behavior, indicating overfitting under the current sample size. Therefore, these exploratory results were not used as primary evidence for biomarker interpretation and are provided only as Supplementary Figure 2 and Supplementary Table 3.

## 4 Discussion

This study represented the first systematic investigation of Mongolian Anda therapy for depression treatment. By integrating 16S rRNA sequencing and metabolomics analyses, we comprehensively characterized microbial composition and metabolite changes between improved and unimproved groups. Our findings identified key bacterial taxa and metabolic pathways (particularly purine/pyrimidine metabolism) and metabolites associated with therapeutic efficacy, providing clinically actionable insights for optimizing depression treatment strategies. The adoption of these rigorous statistical parameters aligns with recently proposed advanced methodologies for high-dimensional microbiome and metabolomics data integration (Zhao et al., 2022; Zhao et al., 2024).

Although numerous studies have reported the association between gut microbiota and depression, the direction of change in alpha diversity—whether it increases or decreases-remains controversial (Bosch et al., 2022). A systematic analysis of 24 independent cohorts revealed three distinct patterns: the majority (54.2%, 13/24) detected no significant differences between depressed individuals and healthy controls; 33.3% (8/24) reported decreased alpha diversity in depression patients; a small proportion (8.3%, 2/24) observed increased diversity, though only one study reached statistical significance (Liang et al., 2023). Interestingly, 16S rRNA analysis in our study observed lower alpha diversity (as indicated by Shannon, PD whole tree, Chao1, and Simpson indices) in both the CK-improve and Treat-improve groups compared to the CK-unimprove and Treat-unimprove groups, although these differences did not reach statistical significance (Figure 3). This finding aligns with a previous report demonstrating higher gut microbiota diversity in non-responders to selective serotonin reuptake inhibitors (SSRIs), suggesting that reduced microbial diversity may be associated with a better treatment response, including Andai therapy (Jiang et al., 2024). However, our results contrast with another study in which non-responders exhibited significantly lower PD whole tree index than responders during antidepressant treatment (Kurokawa et al., 2021). Larger controlled studies are needed to elucidate the relationship between the microbiome change and depression. Overall, the lack of statistically significant differences in alpha diversity between responders and non-responders—both before and after treatment—is consistent with the majority of research, highlighting the complexity of gut microbiota’s role in antidepressant efficacy.

The fecal microbiota composition of the patients in this study was primarily dominated by the phyla Firmicutes, *Bacteroidota*, Proteobacteria, and *Actinobacteriota*, aligning with findings from previous studies (Liu et al., 2022; Zhong et al., 2022). At the phylum level, the CK-improve and Treat-improve groups exhibited a higher relative abundance of Proteobacteria and a lower proportion of *Bacteroidota*. This finding partially corroborated previous reports associating elevated Proteobacteria and *Bacteroidota* levels with depression (Jiang et al., 2015; Yang et al., 2020), with reduced *Bacteroidota* in improved groups. At the genus level, improved patients following Andai therapy showed a marked increase in the relative abundance of *[Eubacterium]_coprostanoligenes_group*, *Bifidobacterium*, *Escherichia-Shigella*, *Faecalibacterium*, and *Dialister*, while genera such as *Klebsiella*, *Prevotella*, *Bacteroides*, and *Parabacteroides* exhibited a declining trend (Figure 8B). These shifts were consistent with existing literature reporting that depression patients typically displayed an enrichment of pathogenic bacterial genera (e.g., *Escherichia*/*Shigella*, *Prevotella, Klebsiella*, *Bacteroides*, *Streptococcus*) and a reduction in beneficial genera (e.g., *Bifidobacterium*, *Faecalibacterium*, *Dialister*, *[Eubacterium]_coprostanoligenes_group)* (Liu et al., 2016; Liu et al., 2022; Quévrain et al., 2016; Samara et al., 2022; Zhong et al., 2022). The findings support the inflammatory hypothesis of depression via gut-brain axis mechanisms (Liu et al., 2020). Pathogenic bacteria may promote neuroinflammation through increased gut permeability and LPS release, while beneficial bacteria enhance anti-inflammatory responses via immunomodulation and barrier protection (Caso et al., 2021; Liu et al., 2020; Liu et al., 2022; Quévrain et al., 2016). The microbial alterations observed in the Anda therapy-improved group generally opposed the dysbiotic patterns typically associated with depression, suggesting that the therapeutic effects of Anda may be mediated, at least in part, through gut microbiota modulation. This interpretation was further supported by reanalysis of an independent public MDD dataset, which showed broadly similar directional changes in several key genera. Although preliminary, this external consistency suggests that the microbiota features associated with Andai response may reflect, at least in part, a more general depression-related microbial pattern.

Notably, we noted some unexpected trends, such as the enrichment of *Escherichia-Shigella*—a pro-inflammatory pathogen known to release LPS that can exacerbate intestinal injury, increase blood-brain barrier permeability, and trigger neuroinflammation (Banks et al., 2015). While this genus is usually positively correlated with depression, its elevation in improved patients implies that the role of gut microbiota in depression may not be strictly dichotomous. Therefore, microbial balance and functional interactions may play a more critical role in disease modulation than the absolute abundance of any single bacterial group.

Of particular interest, *[Eubacterium]_coprostanoligenes_group* demonstrated particular clinical relevance, as its abundance has been previously reported to negatively correlate with depressive symptoms (Liu et al., 2020). This association may be mediated through its cholesterol-modulating function, given that hypocholesterolemia constitutes an established risk factor for depression (Kim and Myint, 2004). Neuroactive metabolites from depression-linked gut microbiota modulate gut-brain axis communication, thereby contributing to depressive pathophysiology (Li et al., 2022).

In our study, we identified significant correlations between *[Eubacterium]_coprostanoligenes_group* abundance and key metabolites including 3’-AMP, cytosine, and 2’-deoxycytidine-5’-monophosphoric acid. Although the *[Eubacterium*]_coprostanoligenes_group was initially discovered to be associated with lipid metabolism, its association with nucleotide metabolites suggests a more complex biological mechanism. Currently, there is no direct evidence indicating that the *[Eubacterium]_coprostanoligenes_group* directly influences nucleotide metabolism. However, there may be multiple connections between lipid and nucleotide metabolism. Firstly, both lipids and nucleotides are essential components of biological membranes, and their synthesis and metabolism may be regulated by common mechanisms. Secondly, certain lipids, such as phospholipids, serve as precursors or cofactors in nucleotide synthesis. For instance, phosphatidic acid is a key intermediate in the *de novo* synthesis pathway of nucleotides. Additionally, the energy produced from lipid metabolism may affect the energy status required for nucleotide synthesis. This opens up new potential research directions for the treatment and clinical detection of depression. However, we also believe that some necessary experiments are required in subsequent studies, such as expanding the sample verification species and investigating the correlation between metabolites and depression, as well as isolating this bacterium *in vitro* to validate its therapeutic effects in mouse models of depression. From a clinical perspective, the most relevant implication of these findings is that treatment-associated microbial and metabolic changes were not isolated events, but converged on a relatively coherent response-related signature centered on nucleotide, purine, and pyrimidine metabolism. In particular, taxa such as *[Eubacterium]_coprostanoligenes_group*, *Escherichia-Shigella*, *Bacteroides*, and *Parabacteroides*, together with metabolites including cytosine, 2’-deoxyadenosine, and 3’-AMP, may serve as candidate biomarkers for distinguishing responders from non-responders and for monitoring treatment-related biological changes. Although these findings are not yet sufficient for clinical application, they provide a basis for future response stratification, longitudinal monitoring, and mechanistic validation in larger cohorts.

Despite revealing the associations between gut microbiota-metabolite profiles and Andai therapy response, this study has several limitations that should be considered. First, the relatively small sample size in each group may affect the statistical power and generalizability of our findings, necessitating validation in larger cohorts. Second, although we controlled for some important demographic variables (age, sex, education, and ethnicity), unmeasured confounders including genetic predisposition, lifestyle factors (e.g., smoking and dietary habits), medication history, and family psychiatric history may influence the results and require future investigation. Thirdly, our analysis primarily focused on the changes in metabolites and metabolic pathways, while the plausible mechanisms remain unexplored. Moreover, the present study was observational in nature and did not include independent validation cohorts or mechanistic experiments; therefore, the identified microbiota–metabolite changes should be interpreted as associations with treatment response rather than direct causal drivers of symptom improvement. Additionally, our current findings were mainly from 16S rRNA sequencing and metabolomics. Integrating multi-omics analyses, such as metagenomics, transcriptomics, and proteomics, can comprehensively uncover the mechanisms by which gut microbiota influences metabolite diversity and depression treatment. Advanced deep learning methods may further improve multi-omics integration and biomarker discovery in microbiota–gut–brain axis research. However, our pilot transformer-based exploratory analysis showed rapid overfitting under the current sample size and was therefore not included as a main result. Larger cohorts will be needed to properly evaluate such models in future studies. Furthermore, the application of sophisticated machine learning models for clinical data analysis, as highlighted in recent literature (Li et al., 2025; Wei et al., 2026), provides a valuable perspective for refining these microbial and metabolic signatures into predictive tools, thereby complementing our current findings and strengthening the overall clinical utility of Andai therapy. In addition, this study did not include direct comparisons with standard antidepressant treatments or other recently proposed therapeutic models for depression, and thus the relative clinical advantage of Andai therapy cannot yet be determined. Future studies with larger cohorts, comprehensive confounder assessments, mechanistic validation experiments, and direct comparisons with contemporary depression treatments will be needed to clarify the causal relevance and clinical positioning of Andai therapy in depression.

## Data Availability

The datasets analyzed during this study are available in the China National GeneBank DataBase (CNGBdb) repository under accession number CNP0009521 at https://db.cngb.org/data_resources/project/CNP0009521/.

## Footnotes

1
https://www.arb-silva.de/

2
https://unite.ut.ee/

3
http://www.hmdb.ca/

4
https://lipidmaps.org/databases

5
https://metlin.scripps.edu/

## Supplementary material

Supplementary Figure 1 External validation of key gut microbial genera in the public 16S rRNA dataset PRJNA591924. Relative abundances of selected genera were compared between healthy controls (HC) and patients with major depressive disorder (MDD). *Indicates a statistically significant difference between the MDD and healthy control (HC) groups (*p* < 0.05).

Supplementary Figure 2 Exploratory transformer-based analysis of microbiome data. **(A)** Training and validation loss curves and validation metrics during model training. **(B)** Validation performance shown by regression and classification results. **(C)** Top 20 feature importance and sample importance distribution derived from the model.

Supplementary Table 1 Demographic and clinical characteristics of the 20 enrolled patients with depression. Sex, age, ethnicity, education level, disease severity, treatment response, and sample collection information for the enrolled patients. CK and Treat indicate pre-treatment and post-treatment samples, respectively.

Supplementary Table 2 LOOCV-based stability analysis of genus-level feature importance in Random Forest models. Mean MDA, Mean MDG, mean ranking positions, and the frequencies of being ranked among the top 10 or top 20 features across LOOCV iterations are shown for each genus in the CK and Treat models. Higher ranking frequency and lower mean rank indicate greater feature stability.

Supplementary Table 3 Prediction summary and feature importance from the exploratory transformer-based analysis. The table includes sample-level prediction results and feature importance scores generated by the TRPCA model.
