# Supplementary figures and images for: Therapeutic impact of Mongolian Andai therapy on gut microbiota and metabolomic profiles in depression
Source: Front Neurosci. 2026 Jun 3;20:1743844. doi: 10.3389/fnins.2026.1743844 (PMC13272016; doi:10.3389/fnins.2026.1743844)

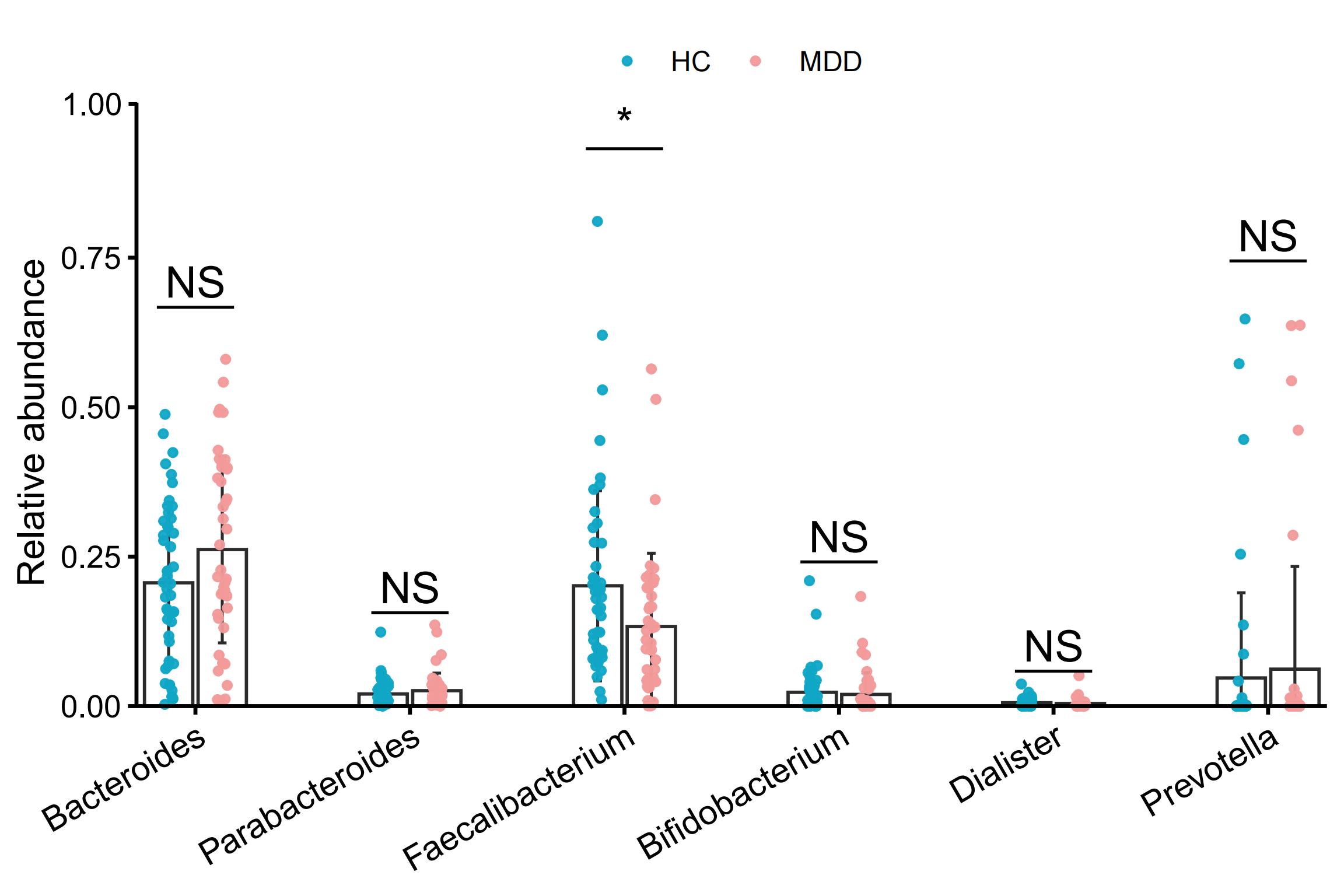

Supplement: Supplementary Figure 1 — External validation of key gut microbial genera in the public 16S rRNA dataset PRJNA591924. Relative abundances of selected genera were compared between healthy controls (HC) and patients with major depressive disorder (MDD). *Indicates a statistically significant difference between the MDD and healthy control (HC) groups (p < 0.05). [file Image_1.jpeg]

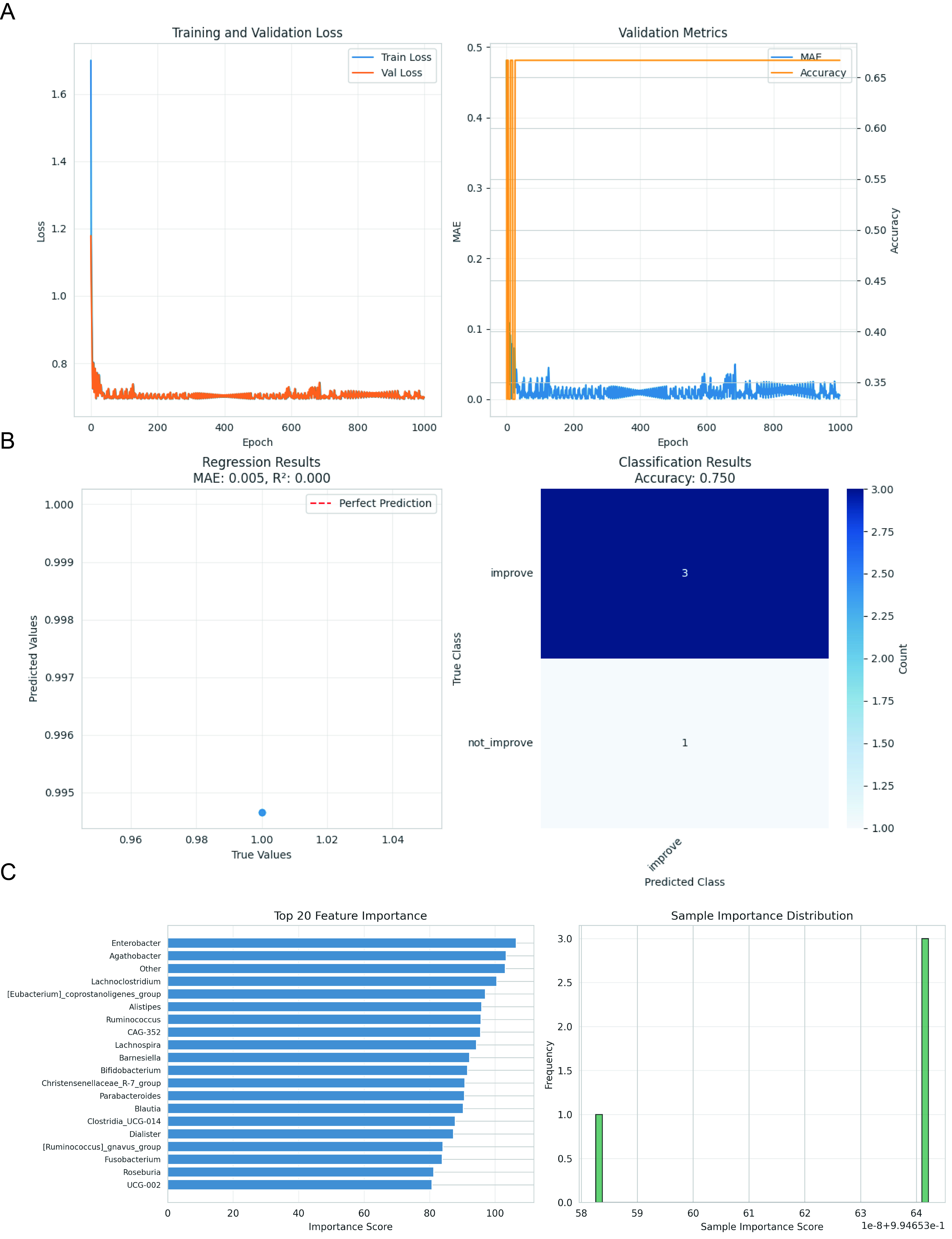

Supplement: Supplementary Figure 2 — Exploratory transformer-based analysis of microbiome data. (A) Training and validation loss curves and validation metrics during model training. (B) Validation performance shown by regression and classification results. (C) Top 20 feature importance and sample importance distribution derived from the model. [file Image_2.jpeg]
